# Taxonomic and functional diversity covary in rock pool microalgal communities despite their different drivers

**DOI:** 10.1002/ece3.7953

**Published:** 2021-07-29

**Authors:** Sonja Aarnio, Janne Soininen

**Affiliations:** ^1^ Department of Geosciences and Geography University of Helsinki Helsinki Finland

**Keywords:** brackish, diatom, diversity index, environmental, functional guild, trait

## Abstract

Local biodiversity has traditionally been estimated with taxonomic diversity metrics such as species richness. Recently, the concept of biodiversity has been extended beyond species identity by ecological traits determining the functional role of a species in a community. This interspecific functional diversity typically responds more strongly to local environmental variation compared with taxonomic diversity, while taxonomic diversity may mirror more strongly dispersal processes compared with functional metrics. Several trait‐based indices have been developed to measure functional diversity for various organisms and habitat types, but studies of their applicability on aquatic microbial communities have been underrepresented. We examined the drivers and covariance of taxonomic and functional diversity among diatom rock pool communities on the Baltic Sea coast. We quantified three taxonomic (species richness, Shannon's diversity, and Pielou's evenness) and three functional (functional richness, evenness, and divergence) diversity indices and determined abiotic factors best explaining variation in these indices by generalized linear mixed models. The six diversity indices were highly collinear except functional evenness, which merely correlated significantly with taxonomic evenness. All diversity indices were always explained by water conductivity and temperature–sampling month interaction. Taxonomic diversity was further consistently explained by pool distance to the sea, and functional richness and divergence by pool location. The explained variance in regression models did not markedly differ between taxonomic and functional metrics. Our findings do not clearly support the superiority of neither set of diversity indices in explaining coastal microbial diversity, but rather highlight the general overlap among the indices. However, as individual metrics may be driven by different factors, the greatest advantage in assessing biodiversity is nevertheless probably achieved with a simultaneous application of the taxonomic and functional diversity metrics.

## Graphical abstract

Functional traits determining the role of a species in a community typically respond more strongly to local environmental variation than conventional taxonomic diversity metrics. We examined the drivers and covariance of taxonomic and functional diversity in coastal microalgal communities by three taxonomic and three functional diversity indices. Despite rather similar performance in explaining local diversity, the individual indices were occasionally driven by different environmental factors; thus, the greatest advantage in assessing local biodiversity is probably achieved with a simultaneous application of both diversity metrics.

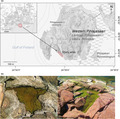

## 1 INTRODUCTION

Biodiversity patterns have been at the center of ecological research for decades. Traditionally, local biodiversity has been quantified with taxonomic diversity metrics such as species richness *S* (the total number of species in a community), Shannon's diversity *H* (accounting both species richness and abundance in a community; Shannon, 1948), or Pielou's evenness *J* (the equality in abundance between species in a community constrained between 0 and 1; Pielou, 1966). Together with abiotic factors, these diversity components are likely to influence ecosystem functioning in different ways (Cardinale et al., 2009; Downing & Leibold, 2002; Lewandowska et al., 2016). For example, temporal and spatial variation in nutrient supply and external salinity stress affect ecosystems both directly and indirectly through species richness, evenness, and diversity (Cardinale et al., 2009).

Despite the indisputable role of these taxonomic diversity indices and their dominant usage in explaining patterns of local biodiversity (Cadotte et al., 2011), ecosystem functions such as productivity are extensively related to inherited traits determining the performance and functional role of a species in a community (Mason et al., 2005; McGill et al., 2006; Villéger et al., 2008). These physiological, morphological, and metabolic traits shared by polyphyletic groups of species typically respond more strongly to local environmental variation than conventional and more complex taxonomic diversity metrics (Mouchet et al., 2010; Reynolds et al., 2002; Rimet & Bouchez, 2012). Especially, variation in microbial traits may largely overweigh taxonomic diversity (Green et al., 2008).

Recently, several trait‐based indices have been developed to measure the three components of functional biodiversity (Hooper et al., 2005; Mouchet et al., 2010; Villéger et al., 2008). Functional richness (*FRic*) measures the amount of niche space filled by species in a community, while functional divergence and functional evenness (*FDiv* and *FEve*, respectively; constrained between 0 and 1) describe the disparity and regularity of species abundance distribution, respectively, in a volume of filled niche space in the community (Villéger et al., 2008). Low functional diversity reduces productivity and biodiversity functioning either through unutilization of available resources (*FRic*) or occupied niche space (*FEve*) in a community, or intense competition for resources through weakly differentiated niches (*FDiv*) (Mason et al., 2005). Together, these three indices quantify trait‐level responses to environmental variation on a continuous scale, unlimited by the high level of detail required for taxonomic species identification (McGill et al., 2006; Violle & Jiang, 2009; Violle et al., 2014).

While these three independent yet complimentary functional diversity indices are successful in explaining diversity patterns and ecosystem functioning for many types of natural communities (Mouchet et al., 2010), their overall feasibility in coastal microbial communities has remained understudied (Alahuhta et al., 2018; Schmera et al., 2016; Villéger et al., 2008). Future climate‐driven changes in sea level and precipitation and consequent alterations in nutrient and salinity conditions especially in the northern latitudes pose a major threat on the unique brackish biota along the marine‐freshwater transition zone of the brackish Baltic Sea (Flöder et al., 2010; Hernando et al., 2015). Diatoms are important players in biomass production in coastal rock pools, strongly influenced by environmental variation. Majority of rock pool diatoms are ecological specialists, highly adapted for the harsh pool environment by various traits (Jocque et al., 2010). Isolated rock pools comprising diverse diatom communities offer an interesting setting for testing the influence of environmental stress on microbial diversity and ecosystem properties in the era of global climate change over different timescales (Blaustein & Schwartz, 2001; Srivastava et al., 2004).

Our study aimed first to investigate how and to what extent the taxonomic diversity covaries with the functional diversity among these communities. We hypothesize that (H_1a_) the taxonomic and functional diversity metrics are significantly correlated with each other, yet (H_1b_) the strength of correlation varies among the indices (Mouchet et al., 2010; Schmera et al., 2016). Secondly, we examine which environmental variables best explain diatom taxonomic and functional alpha diversity among coastal rock pool communities. We hypothesize that (H_2a_) the effects of local environmental variation on diatom diversity are better captured by the functional diversity indices compared with the taxonomic ones due to ecological adaptations, while (H_2b_) taxonomic diversity indices relate more strongly with spatial gradients than functional diversity indices as taxonomic metrics respond more to dispersal processes (Erős et al., 2009; Heino, 2008; Leibold & Chase, 2017).

## 2 MATERIAL AND METHODS

### 2.1 Field sampling

**FIGURE 1:**
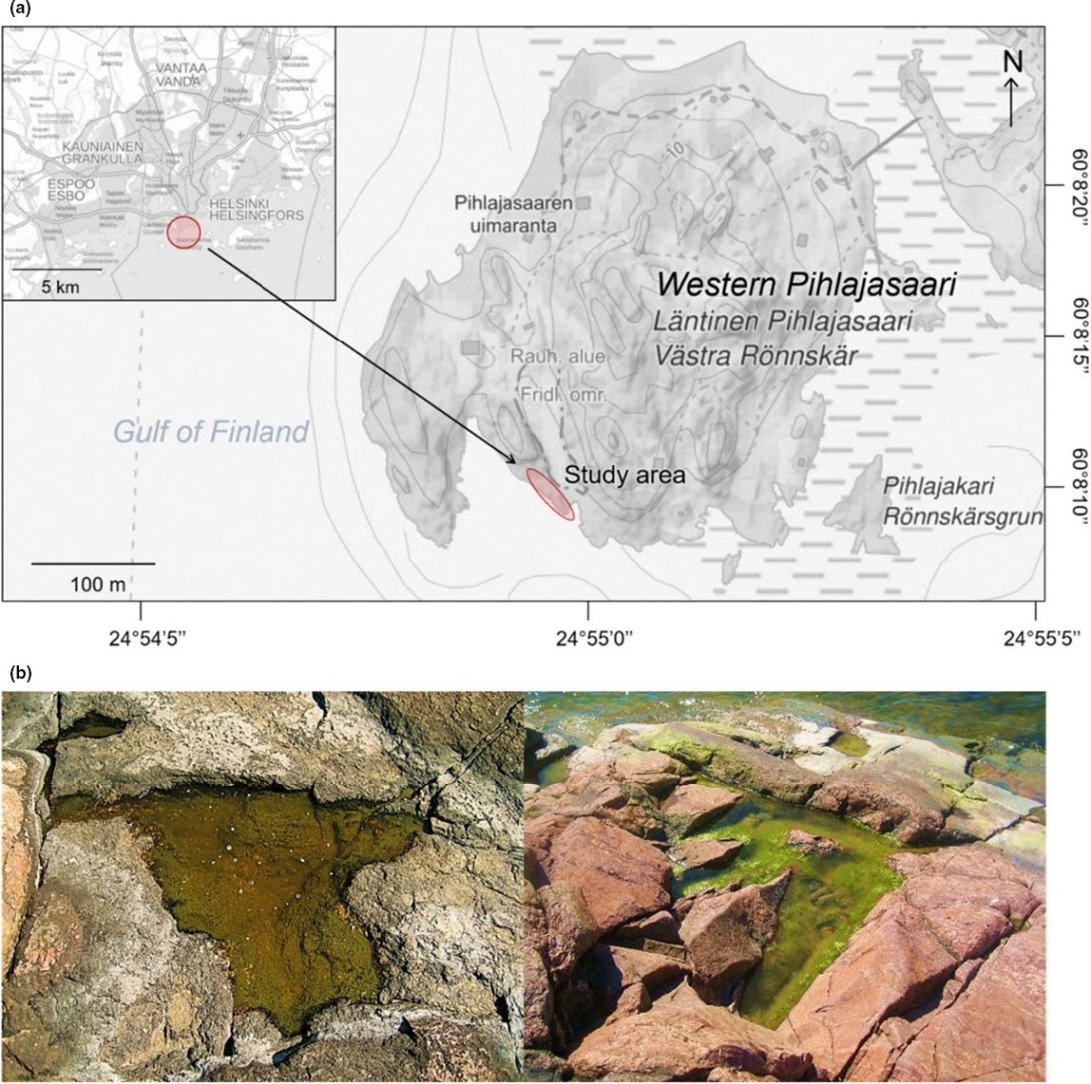
(a) Map of the study area. The study area is located on the western Pihlajasaari island c. 2 km off the coast of Helsinki (red circle on the index map) on a rocky outcrop on the southwestern part of the island (red oval on the larger map), surrounded by the Gulf of Finland, the northern part of the Baltic Sea. (b) Some of the sampled rock pools on the western Pihlajasaari Island on the Baltic Sea coast. Pool 5 (left) characterized by shallow (0.15 m), clear water, and visible bottom sediment, and pool 25 (right) located at an immediate proximity to the sea with an exceptionally large coverage of macroalgae. Photos: Sonja Aarnio; map: National Land Survey of Finland (2018)

We sampled 30 brackish‐watered, isolated rock pools once a month (17 May, 22 June, and 22 July) in 2016 on a granitic outcrop in the western island of Pihlajasaari (66°68′449″N, 38°40′48″E), ca. 2 km south of Helsinki on the coast of the northern Baltic Sea (Figure 1). The studied rock pools were mainly rainfall‐fed, yet the pools closest to the sea were influenced by the brackish seawater by wind‐caused waves and salty sprays from the sea. Nutrient enrichment was likely mostly of biological origin, caused by decaying organism remains, leaf litter, and faunal excretions such as bird droppings (Brendonck et al., 2016; Methratta, 2004). None of the pools were shaded by the sparse aquatic and terrestrial vegetation allowing light to penetrate deep toward the pool base, making solar radiation conditions comparable (Hill, 1996).

To minimize the effects of diurnal variation on water physicochemistry such as thermal stratification, we sampled the pools roughly at the same time of the day each month (Ganning, 1971). We measured water pH, conductivity, and temperature in the field with YSI field meter, and pool morphometrics (i.e., max depth, length, and width) with a meter stick to the nearest centimeter, and calculated pool area (length * width). We collected a 0.5 L water sample from each pool in a plastic container preserved at 4℃ until the determination of total P (SFS‐EN ISO, 2004) and total N (SFS‐EN ISO, 1998) concentration in a laboratory. The determination of total N was occasionally disturbed by high organic matter content, which, like nitrate ions (NO_3_−) in nitrogen compounds, also absorbs radiation on the UV wavelength of 220 nm measured by the UV/VIS spectrometer. Due to such a high disturbance in 21 water samples (15 in June and 6 in July) and two results with concentrations exceeding the determination limit in May, we omitted all total N values from the data.

Due to the small spatial scale and consequent difficulties in determining the exact pool coordinates by GPS or from aerial photographs, we estimated pool X and Y coordinates (based on the perpendicular pool distance from the shore and the horizontal pool distance from the map origin in the southern end parallel to the shoreline, respectively) and mean isolation as a mean Euclidean distance (i.e., the sum of distances to five closest pools divided by five; Vanschoenwinkel et al., 2007) for each pool from a drawn grid map of the study area showing the relative location of the sampled pools to each other and to the seashore (Appendix 1). In our data analyses, we used these estimated spatial metrics instead of spatial filters (e.g., Moran eigenvector maps) as in our study setting, X and Y had very different nature (see above) and hence could not be combined rationally into spatial filters.

### 2.2 Taxonomic data

We sampled benthic diatoms following EN 13946 standard (2003) by collecting ten epilithic subsamples (ca. 25 cm^2^) from each pool bottom with a toothbrush and combined these as a single composite sample in a plastic test tube in the field. From the two steepest‐walled pools (pools 10 and 14) with unreachable bottom sediment, only the pool walls were sampled deep enough to ensure the frustules were permanently submerged. Between each sampling, the toothbrush was rinsed in pool water to remove any attached cells as this effectively reduces the possibility of contamination and taxonomic bias between the samples (Kelly et al., 1998).

The samples were stored at 4℃ for 24 hr until treatment with 30% H_2_O_2_ to remove organic material and mounting on slides with Naphrax. A total of 500 valves per slide were counted and identified to the lowest taxonomic level possible (mostly species level) with a light microscope (1000× magnification) following Krammer and Lange‐Bertalot (1986–1991) and Cantonati et al. (2017). From the four most sparsely celled slides, less than 500 valves could be counted. We created two taxonomic site‐species matrices based on species relative abundances and on binomial (0/1) presence–absence data, respectively.

### 2.3 Functional data

**FIGURE 2:**
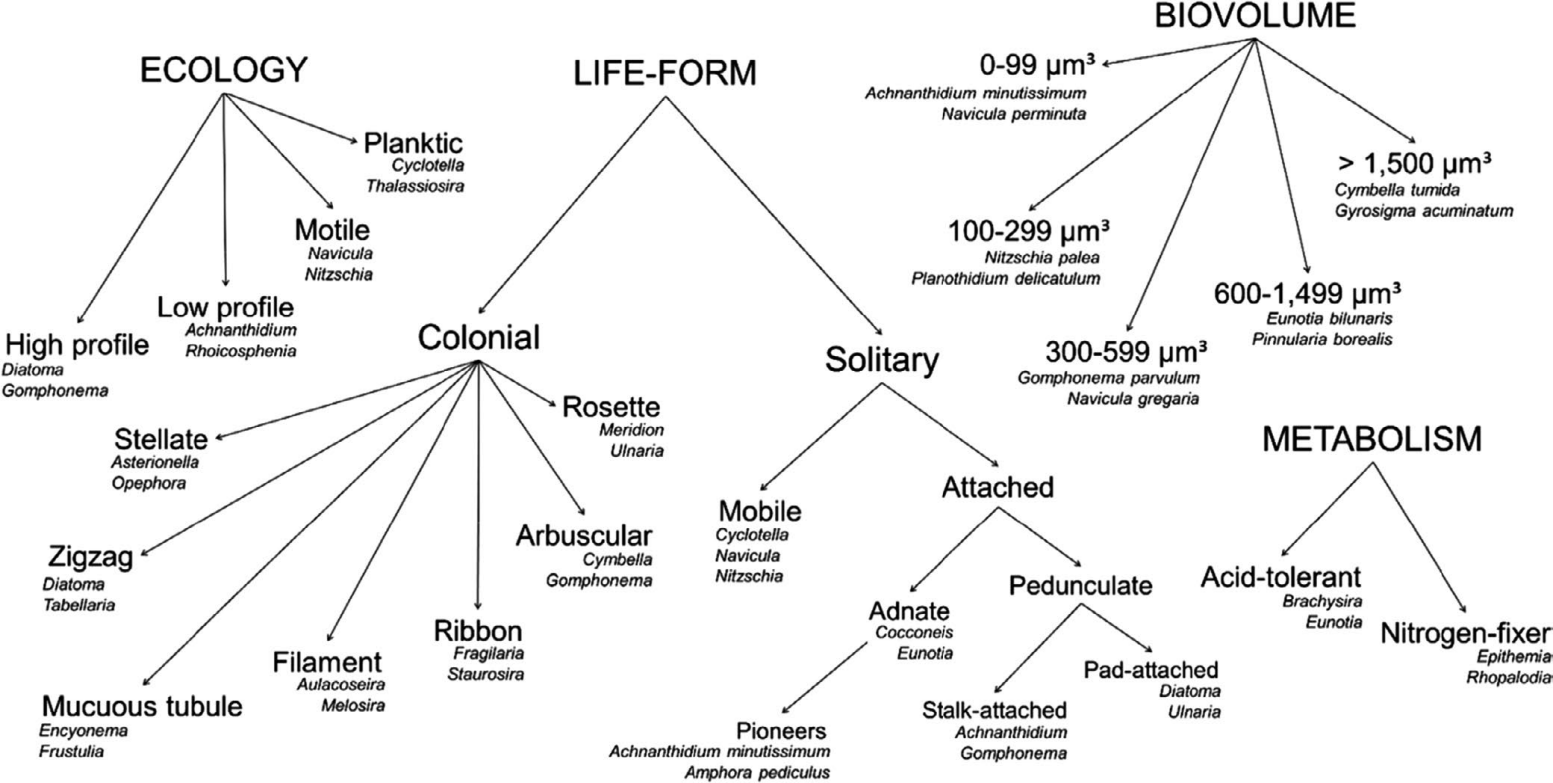
Classification of the diatom taxa after their life form, ecology, metabolism and biovolume into functional guilds after Passy (2007), and Rimet and Bouchez (2012). For each guild, example taxa are also given. Note that some of the guilds are partly overlapping, and a single taxon may be classified into multiple guilds. Freely moving mobile species were first separated from species attached to their substrate; the attached species were further subdivided after the mode of attachment into adnates (attachment by valve face or girdle view) or pedunculates (attachment by mucilage stalk or pad). Additionally, small adnate species *Achnanthidium minutissimum* and *Amphora pediculus* were classified as pioneers (species first to colonize bare substrate). The colonials were classified after the form of colony into mucous tubule (freely moving taxa encapsuled in mucous substance), filament (centrics linked by, e.g., spines), zigzag (mucilage pennates), rosette (stalked pennates), ribbon (pennates attached by valves), stellate (mucilage pennates forming star‐shaped colonies), and arbuscular (taxa forming stalked, branching colonies) guilds. Finally, a separation was made between high profile (tall‐statured, large filamentous, tube‐forming or stalked taxa), low profile (short‐statured slowly moving taxa), motile (mobile fast‐moving taxa), and planktic guilds (mobile floating taxa), and between acid tolerants and nitrogen fixers (see Material and methods for descriptions). The five cell size classes are based on biovolume calculated after cell length, width, thickness, and shape

The identified diatom species were classified into 21 partly overlapping functional groups (Figure 2). We first divided the species into five size classes after their biovolume (determined by cell length, width, thickness, and shape) and 14 life‐form categories after interspecific morphological adaptations to physical and chemical disturbance (i.e., cell motility, posture, and type of adhesion) following Rimet and Bouchez (2012). A single taxon may have various successive life forms and may thus be classified into multiple life‐form categories (Berthon et al., 2011).

We further classified the species after their preferences for nutrient concentration and physical disturbance into three ecological guilds (high profile, low profile, and motile) after Passy (2007), and a fourth guild (planktonic) proposed by Rimet and Bouchez (2012). High‐profile guild forms the upper benthic layer in nutrient‐rich, weakly disturbed habitats, whereas low profile guild dominates highly disturbed and oligotrophic habitats in the base of the benthos. Motile guild consists of nutrient‐tolerant taxa with low tolerance of physical disturbance—yet as physically capable of selecting a suitable habitat, the species are only marginally affected by disturbance and resource limitation—while planktic guild includes species morphologically adapted to lentic, less turbulent environments prone to sedimentation.

Finally, we separated between acid‐tolerant (acidobiontic or acidophilus species with pH optimum <7 in Van Dam et al. (1994) and nitrogen‐fixing species (members of the genera *Denticula*, *Epithemia,* and *Rhopalodia* with cyanobacterial endosymbionts capable of fixing atmospheric nitrogen) (Soininen et al., 2016). We created two data matrices: one for species‐trait data and another for binomial site‐trait data. In the species‐trait matrix, each species belonging to a given guild (other than continuous biovolume guild on a scale 1–5) was given a value of 1; otherwise, the value was set to 0. Each species could be characterized by multiple traits and could thus belong to more than only one guild.

### 2.4 Statistical analyses

Prior to statistical analyses, the distribution of the continuous explanatory variables was examined for outliers with a Cleveland dot plot (Cleveland, 1993), and the variables with clearly deviating values were ln‐transformed to reduce their skewness. We calculated Spearman's rank correlation coefficients and variation inflation factors (VIF; Marquardt, 1970) with a threshold value of 5.0 (Zuur et al., 2010) to detect any statistical dependence between the variables. Hence, Y coordinates and all pool morphometrics other than water depth were excluded from the regression models. We examined temporal and spatial patterns in the data with coplots and potential three‐way interactions between each response and continuous covariate by fitting 3 monthly bivariate linear regression models in a multipanel coplot.

We quantified species richness (hereafter *S*), Pielou's evenness (*J*), and Shannon's diversity (*H*) for the taxonomic relative abundances. We further quantified functional richness (*FRic*) applying the convex hull volume index, functional evenness (*FEve*) using the minimum spanning tree (MST), and functional divergence (*FDiv*) with the center of gravity of the convex hull vertices. All indices were calculated using the first two ordination axes of principal coordinate analysis (PCoA; Gower, 1966) for the square root corrected species–species distance matrix; *FEve* and *FDiv* were further weighted with the species relative abundances (Villéger et al., 2008).

We used generalized linear mixed models, or GLMMs (Zuur et al., 2013), to examine the most influential factors explaining variation in the six diversity indices. Along with the continuous explanatory variables, we created two categorical covariates: one for the sampling site (ranging from 1 to 30) and one three‐level covariate for the sampling month (Wood, 2006). Since each site was sampled three times, that is, once a month (Appendix 2), we treated sampling month as a fixed term and sampling site as a random intercept (Pinheiro & Bates, 2000; Zuur et al., 2010). For the explanatory variables with a clearly unimodal relationship with the response variables, quadratic terms were used; otherwise, we used only first level terms. We also included two‐way interactions between sampling month and each physicochemical covariate to account for temporal variation in the X–Y relationship over the 3 months. Prior to the GLMMs, all the continuous covariates were standardized. We decided to remove all data for pool 3 in July from the functional GLMMs due to exceptionally low functional diversity, as representing such a clear outlier strongly influenced the resulting models for the three functional indices.

We run the full models either with Poisson (for *S*) or Gaussian error distribution (for the rest of the diversity indices). Due to a slight overdispersion in the residuals of the reduced Poisson GLMM, we refitted the model with a negative binomial error distribution. The covariates were removed from the full GLMMs by a backward stepwise method, and the model with the lowest AIC value (Akaike information criterion; Akaike, 1973) was considered the best. The statistical significance and explanatory power of the covariates in the reduced GLMM models were assessed by the likelihood ratio test (LRT) and Nakagawa's *R* ^2^ (Nakagawa & Schielzeth, 2012), respectively.

All models were validated following the protocol proposed by Zuur et al. (2013), and Zuur and Ieno (2016). Since the goodness of the stepwise selected ‘best’ approximating model is always relative to the variables chosen to be included in that model from all possible, potentially competing subsets of the full model (Whittingham et al., 2006), we assessed model independence by plotting the residuals against the fitted values, and the covariates both included in, and excluded from each of our models. We checked the significance of any nonlinear pattern between the model residuals and a covariate with a generalized additive model (GAM; Hastie & Tibshirani, 1990). We assessed spatial independence of the residuals by Moran's *I* (Moran, 1950) using Bonferroni‐corrected *p* values for the correlation coefficients (Legendre & Legendre, 2012).

All statistical analyses were conducted with R (version 3.6.2; R Core Team, 2020) by utilizing an R source code provided by Zuur et al. (2009) with packages listed in Appendix 3.

## 3 RESULTS

The studied pools were generally small (area range 0.1–36.7, mean 3.8 m^2^), shallow (depth range 0.05–0.4, mean 0.2 cm), eutrophic (total P range 17.3–1,448.3, mean 214 μg/L), and alkaline (pH range 6.7–10.0, median 8.6), with high conductivities (range 38–16,741; mean 4,101 μS cm^−1^) (Appendix 4; see Aarnio et al., 2019 for more detailed description). The pools followed a clear negative gradient in water conductivity (*r*_s_ = −0.5) and pool depth (*r*_s_ = −0.3) along increasing distance to the sea. Water conductivity covaried positively with pH (*r*_s_ = 0.2) and negatively with temperature (*r*_s_ = −0.3), the latter scaling positively with total P concentration (*r*_s_ = 0.5). Total P (*r*_s_ = −0.3), pH (*r*_s_ = −0.6), temperature (*r*_s_ = −0.4), and pool depth (*r*_s_ = 0.2) were further significantly related to sampling month (Appendix 5).

A total of 179 species (site range 5–52, mean 31) belonging to 62 genera were recorded. Most of the species were rare and only sporadically present in low abundances. The majority of the species were mobile (81% of all 179 species) and solitary (80%), while a fair half (53%) belonged to the motile guild. Few species (3%) were classified as nitrogen fixers, while roughly 11% were acid‐tolerant (Appendix 6). The most abundant guilds—that is, solitary (90%), mobile (84%), and motile (55%)—were also spatially widespread, while the least diverse stellate and rosette colonials (< 1%) were rare and highly sporadic by presence. On the contrary, species‐poor zigzag colonials were consistently present in at least 80% of the pools despite low abundance (monthly mean ≤8%). The size distribution was nearly equal, with 66% of the species belonging to size classes 2–4 (22% in each) and the remaining 34% in classes 5 (18%) and 1 (15%).

**FIGURE 3:**
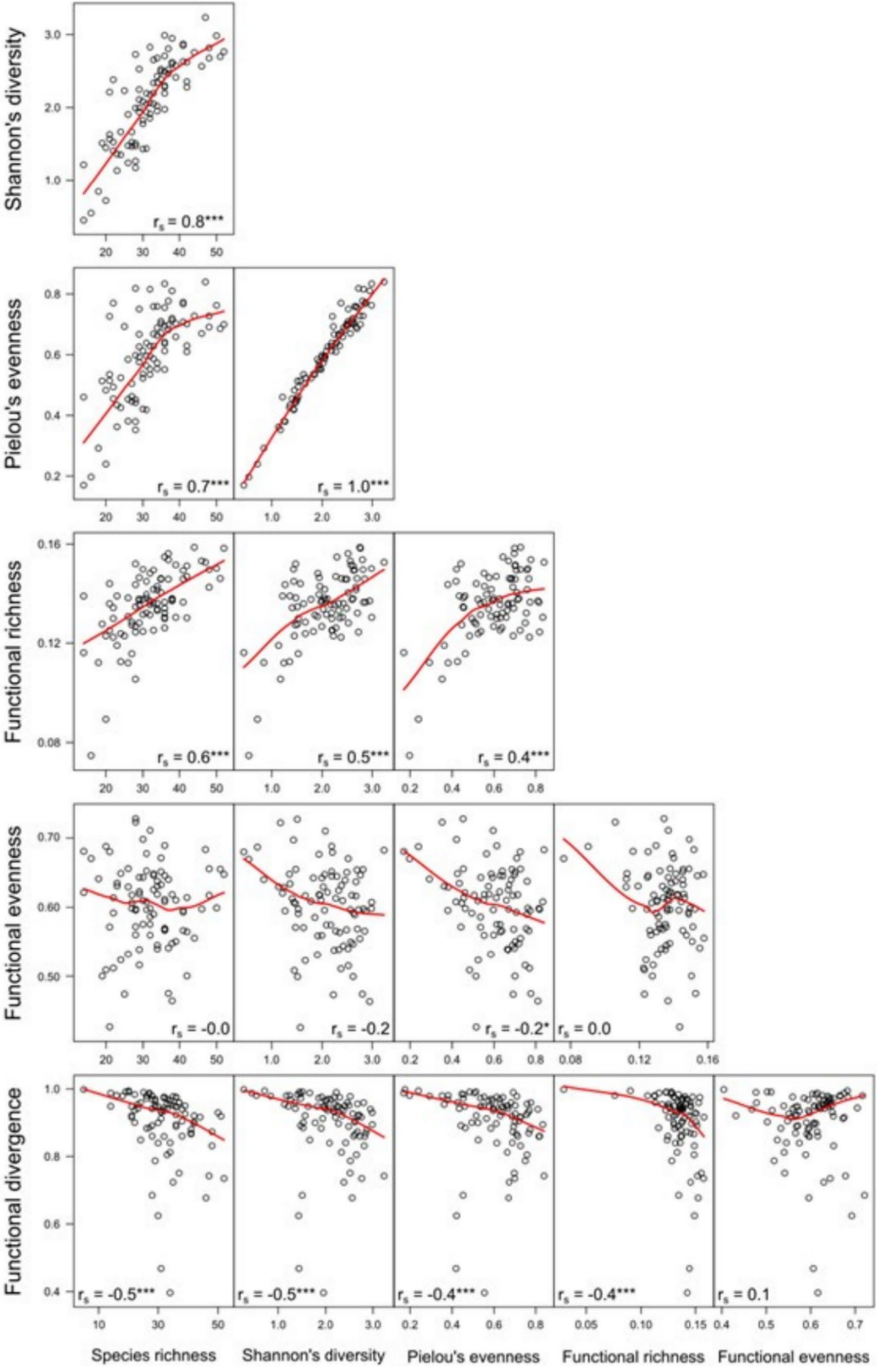
Pairwise correlations between the six diversity indices represented by a fitted Lowess smoother and Spearman rank correlation coefficients. The statistical significance of correlations is based on the *p* value: *p < .05, **p < .01, ***p < .001

The abundances were occasionally relatively evenly distributed in both taxonomic (*J*_range_ 0.2–0.8, *J*_mean_ 0.6) and functional space (*FEve*_range_ 0.4–0.7, *FEve*_mean_ 0.6) (Table 1). On average, *FDiv* was considerably high (range 0.4–1.0, mean 0.9) in our study system. Spatial variance in both taxonomic and functional diversity peaked in July; the monthly diversity range was always widest for *FDiv* ja shortest for *FRic* (range_May‐July_ 0.03–0.16, mean 0.13). Temporal variation in all six diversity indices was distinctly high in pool 3, and in pool 17 for *J* and *H* (range_May‐July_ 0.3–3.2, mean 2.1) (Appendix 7). Significant positive pairwise correlation was found between all diversity indices (*r*_s_ > 0.4) except for *FDiv*, which was negatively related to the other indices (*r*_s_ > −0.4), and for *FEve*, which correlated significantly only with *J* (*r*_s_ = −0.2) (Figure 3).

**TABLE 1:** Statistics and performance of the generalized linear mixed models for the taxonomic and functional diversity indices

Index	Min	Max	Mean	Median	*SD*	AIC	$R_{c}^{2}$	$R_{m}^{2}$
*S*	5	52	31	32	8.72	632.95	0.45	0.41
*H*	0.28	3.23	2.06	2.19	0.63	130.05	0.57	0.53
*J*	0.17	0.84	0.59	0.62	0.15	−119.69	0.51	0.50
*FRic*	0.03	0.16	0.13	0.14	0.02	−526.45	0.33	0.33
*FEve*	0.40	0.72	0.60	0.61	0.06	−257.18	0.47	0.26
*FDiv*	0.40	1.00	0.90	0.94	0.11	−176.30	0.52	0.47

Note

The model selection was based on the Akaike information criterion; the explanatory power of the entire model and the fixed effects was assessed by conditional and marginal Nakagawa's *R* ^2^ values, respectively.

**FIGURE 4:**
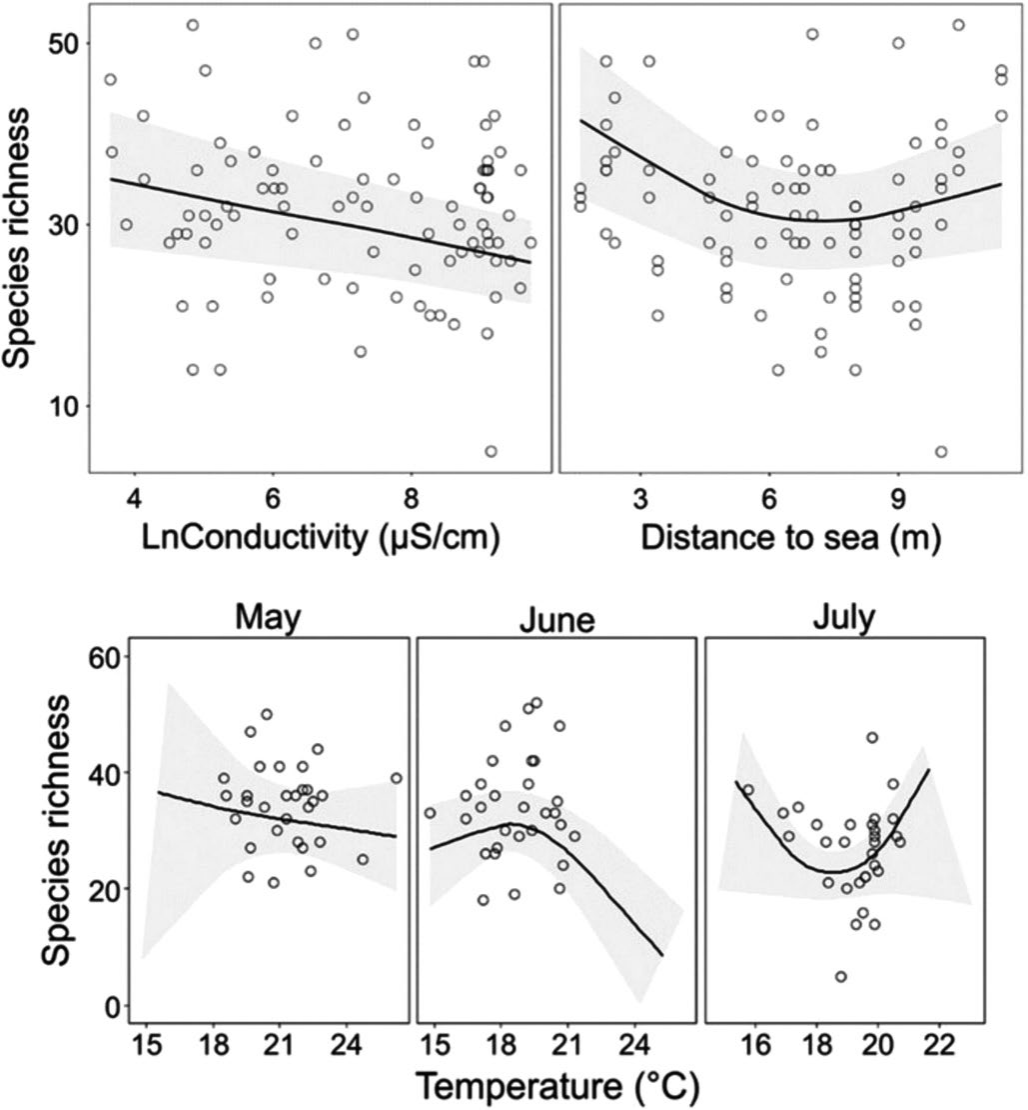
Fit of the generalized linear mixed models for species richness. Shown is the model fit for statistically significant covariates explaining variation in the response variable, including a fitted Lowess smoothing curve with 95‐% confidence intervals (the gray area). The hollow circles represent the observed relationship between the response variable and the covariates. Note that the conductivity values are ln‐transformed. For the rest of the taxonomic indices, see Appendix 8

**FIGURE 5:**
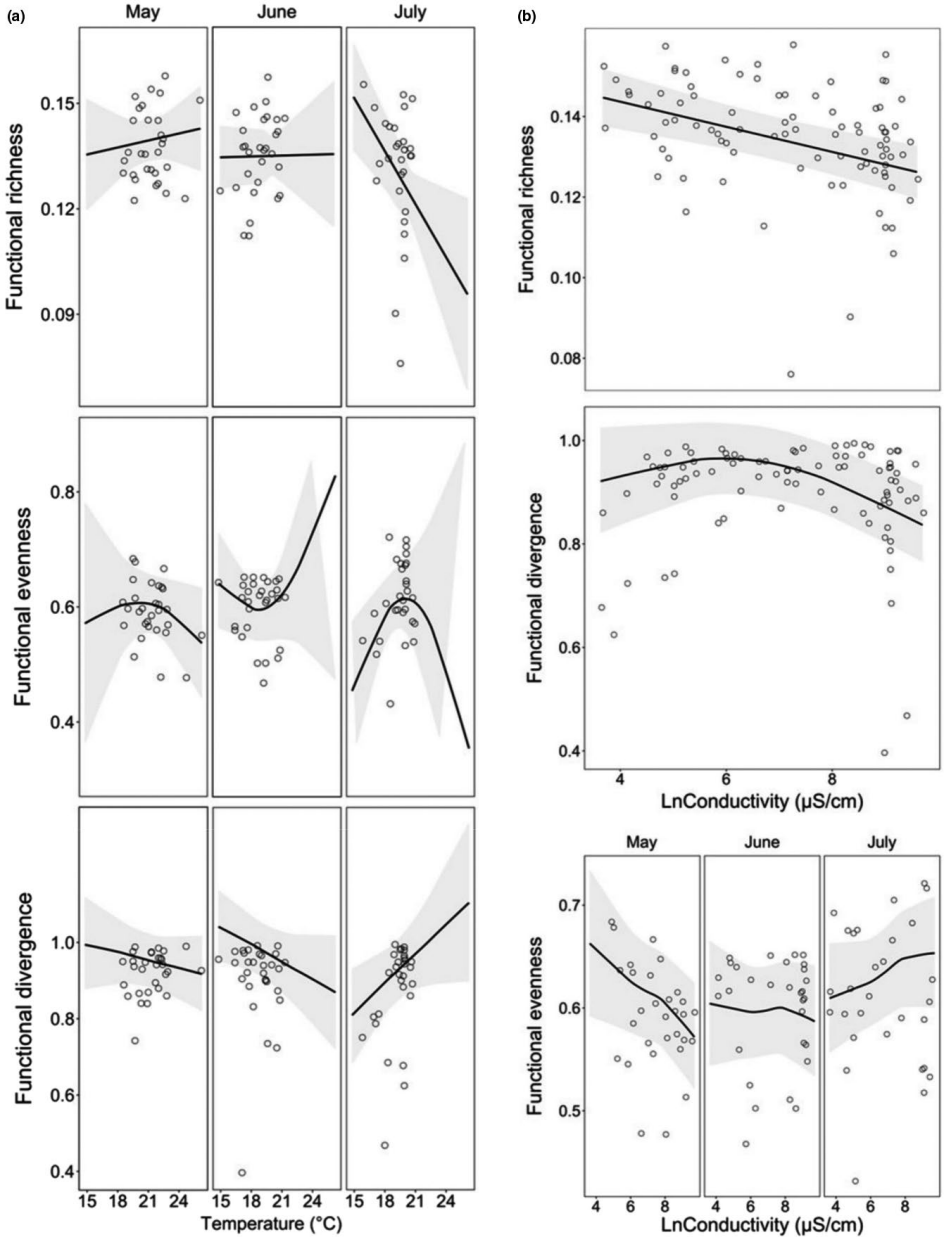
Fit of the generalized linear mixed models for the functional diversity indices. Shown is the model fit for (a) month–temperature interaction and (b) water conductivity explaining variation in the response variable, including a fitted Lowess smoothing curve with 95% confidence intervals (the gray area). The hollow circles represent the observed relationship between the response variable and the covariates. Note that the conductivity values are ln‐transformed. For the rest of the functional indices, see Appendix 9

According to the best approximating GLMMs, variation in the taxonomic diversity indices was always significantly explained by a negative linear relationship with water conductivity (*p* < .05), a U‐shaped relationship with pool distance to the sea (*p* < .05), sampling month (*p* < .001), and the interaction between water temperature and sampling month (*p* < .05) (Tables 2 and 3, Figure 4, Appendix 8). Variation in the functional diversity indices was always significantly explained by the interaction between sampling month and water temperature (*p* < .05), and by water conductivity, the latter either directly (*FRic* and *FDiv*; *p* < .01) or by an interaction with sampling month (*FEve*; *p* < .05) (Figure 5). Both *FRic* and *FDiv* were related to pool isolation (*p* < .05); *FRic* was further explained by pool depth (*p* < .05) and *FDiv* by total P (*p* < .05) and pool distance to the sea (*p* < .001) (Appendix 9).

**TABLE 2:** Results of the generalized linear mixed models for the taxonomic and functional diversity indices

Index	Coefficient	Estimate	*SD*	*df*	LRT	Pr(>Chi)	Significance
*S*	(Intercept)	3.48738	0.06249				
	Conductivity	−0.08668	0.03332	1	6.2297	0.012562	*
	Distance to sea	−0.03984	0.03213	1	1.5148	0.218404	
	Distance to sea^2^	0.08457	0.02457	1	9.9181	0.001637	**
	Temperature:Month			2	6.5813	0.037230	*
	Temperature^2^:Month			2	9.7619	0.007590	**
	Month			2	26.504	0.000002	***
*H*	(Intercept)	2.31718	0.12575				
	Conductivity	−0.20537	0.06443	1	9.5450	0.002005	**
	Distance to sea	−0.16600	0.06489	1	6.3403	0.011802	*
	Distance to sea^2^	0.20111	0.05060	1	12.9696	0.000317	***
	Temperature:Month			2	5.8021	0.054966	
	Temperature^2^:Month			2	11.2059	0.003687	**
	Month			2	38.6000	4.15E−09	***
*J*	(Intercept)	0.66363	0.03124				
	Conductivity	−0.04561	0.01572	1	8.1809	0.004233	**
	Distance to sea	−0.04123	0.01570	1	6.8154	0.009038	**
	Distance to sea^2^	0.04286	0.01205	1	10.7379	0.001050	**
	Temperature:Month			2	3.8752	0.144051	
	Temperature^2^:Month			2	8.4818	0.014395	*
	Month			2	34.7170	2.89E−08	***
*FRic*	(Intercept)	0.13862	0.00277				
	Conductivity	−0.00549	0.00142	1	12.1613	0.000488	***
	Depth	0.00269	0.00133	1	4.0081	0.045283	*
	Isolation	0.00338	0.00130	1	6.5420	0.010536	*
	Temperature:Month			2	6.5949	0.036978	*
	Month			2	9.4052	0.009072	**
*FEve*	(Intercept)	0.60695	0.01261				
	Isolation	−0.01385	0.00702	1	3.6320	0.056680	
	Conductivity:Month			2	8.2812	0.015910	*
	Temperature:Month			2	0.1593	0.923460	
	Temperature^2^:Month			2	6.0408	0.048780	*
	Month			2	7.7345	0.020920	*
*FDiv*	(Intercept)	0.96155	0.02413				
	Total P	0.02779	0.01258	1	4.8147	0.028218	*
	Conductivity	−0.04622	0.01311	1	11.9792	0.000538	***
	Conductivity^2^	−0.03511	0.01256	1	7.4806	0.006237	**
	Distance to sea	−0.05220	0.01297	1	15.2788	0.000093	***
	Distance to sea^2^	−0.01891	0.01001	1	3.3759	0.066156	
	Isolation	−0.03961	0.01067	1	12.5414	0.000398	***
	Temperature:Month			2	6.6338	0.036265	*
	Month			2	1.5475	0.461275	

Note

That estimates and their standard errors are only shown for continuous covariates without interactions. The statistical significance of the covariates is based on the likelihood ratio test and indicated by the *p* value: *p < .05, **p < .01, ***p < .001.

**TABLE 3:** Summary of statistical significance of the explanatory variables for each of the six diversity indices

	*S*	*J*	*H*	*FRic*	*FEve*	*FDiv*
Total P	–	–	–	–	–	*
pH	–	–	–	–	–	–
Conductivity	*	**	**	***	*^a^	***
Temperature^a^	**	*	**	*	*	*
Depth	–	–	–	*	–	–
Distance to sea	**	**	***	–	–	***
Isolation	–	–	–	*	ns	***
Month	***	***	***	**	*	ns

Note

For each explanatory variable, the highest significance level is shown irrespective of the term order. The statistical significance is based on the likelihood ratio test of the generalized linear mixed models and indicated by the *p* value: ns nonsignificant, *p < .05, **p < .01, ***p < .001, – variable not included in the reduced model.

^a^ Interaction with sampling month.

We did not find significant difference in the explained variance for any of our GLMM models when compared to models without the random variable for pool site except for *FEve* ($R_{c}^{2}$ 0.47, $R_{m}^{2}$ 0.26, *p* < .05). For *FRic*, marginal and conditional *R* ^2^ were equal; otherwise inclusion of the random structure slightly increased marginal *R* ^2^ (Table 1). Overall, the explained variance was highest for *H* ($R_{c}^{2}$ 0.57, $R_{m}^{2}$ 0.53) and lowest for *FRic* ($R_{c}^{2}$ and $R_{m}^{2}$ 0.33).

## 4 DISCUSSION

We applied three taxonomic (*S*, *H,* and *J*) and three functional (*FRic*, *FEve,* and *FDiv*) diversity indices to examine environmental variation and consequent differences in diatom diversity among 30 coastal rock pools. Contrary to our primary hypothesis, we could not fully separate between the taxonomic and functional diversity indices in their ability to explain local biodiversity among the microalgal communities. All six diversity indices were always explained by water conductivity and temperature–sampling month interaction. Variation in both taxonomic and functional diversity was further always associated with pool spatial location except for *FEve*, largely disagreeing with our secondary hypothesis as well. While our models succeeded in finding the most significant environmental factors explaining both taxonomic and functional diversity in our study system, the explained variance did not markedly differ between these two sets of regression models. Despite a slight emphasis toward the importance of the physicochemical variables for the functional diversity, our results did not clearly support our hypothesis of an overall superiority of the functional diversity metrics over the taxonomic ones in explaining local environmental conditions.

A clear majority of the studied pool taxa were motile, freely moving noncolonials, followed by pedunculate and high‐profile guilds. This is not unusual given the known dominance of especially motile and high‐profile guilds in disturbed, eutrophic, well‐lit epilithic habitats (Passy, 2007; Reynolds et al., 2002). Promoted by short‐term alterations in local microclimate, frequent physicochemical disturbance characteristic of small coastal waterbodies exposes the biota to highly variable environmental conditions, increasing local diversity by setting demands for highly specialized biota (Ganning, 1971; Metaxas & Scheibling, 1993; Virtanen & Soininen, 2012).

### 4.1 Taxonomic diversity

Diatom diversity is heavily influenced by spatiotemporal variation in local environment, reflected also in the studied communities (Aarnio et al., 2019; Stomp et al., 2011; Verleyen et al., 2009). Variation in taxonomic diversity was consistently significantly explained by water conductivity, sampling month, pool distance to the sea, and an interaction between sampling month and water temperature, regardless of the taxonomic index. The declining diversity along the conductivity gradient, and the U‐shaped relationship with pool distance to the sea is most likely explained by the interplay between these two variables. Water conductivity is an important factor regulating pool biota especially in coastal areas under significant seawater influence, and even minor changes along the conductivity gradient may have major impact on local biodiversity (Vanormelingen et al., 2008). The pools closest to the sea were largely directly contacted with seawater throughout the summer and characterized by relatively cool, alkaline, less nutritious, and highly conductive water typically associated with lower species diversity. Through mass effect, continuous introduction of effectively dispersing species into less environmentally suitable pools may nevertheless enable temporary species occurrence outside their preferred distribution range (Logue et al., 2011), allowing the maintenance of diverse communities under elevated salinities. In turn, eutrophic, warm‐watered, less conductive, and more acidic pools often maintain greater diversity, adding to species diversity of the more inland pools (Aarnio et al., 2019; Soininen et al., 2016; Stenger‐Kovács et al., 2019).

Pool distance to the coastline affected each component of taxonomic diversity, supporting our view of the suitability of the taxonomic indices in capturing spatial control over the communities. The steady decline in taxonomic diversity toward the end of the summer, and the observed monthly variation in the temperature–diversity relationship likely resulted from higher resource similarity due to decreasing variation and strengthening of environmental control, previously observed in a concurrent study within the same pool metacommunity (Aarnio et al., 2019).

### 4.2 Functional diversity

Functional diversity was linked to varying physicochemical variables in our pool communities. Nutrient enrichment increased *FDiv*, while *FRic* declined in shallower pools. *FRic*, *FEve,* and *FDiv* were further reduced in elevated conductivities, yet in July, *FEve* increased along the conductivity gradient. The previously discovered strengthened environmental control during the summer (Aarnio et al., 2019) probably explains the significant negative temporal trend in *FRic*, as intensified resource differentiation further diminishes the occupied trait space. Rather similarly, significant temporal variation in water temperature might well explain the decrease in *FRic* and increase in *FDiv* in warmer pools in May–June and the opposite trend for these indices in July, as well as the temporary change from negative to positive unimodal relationship with *FEve* in June.

While allowing maintenance of several ecological niches through resource differentiation, stressful environmental conditions restrict survival of the most salt‐intolerant and oligotrophic species especially in shallow coastal pools such as studied here (Aarnio et al., 2019; Lewandowska et al., 2016; Mazzei et al., 2018). Less limited by resource competition, the specialized, functionally dissimilar biota are typically clustered in uneven densities toward the edges of more sparsely occupied functional space unfavorable to most of the species (Teittinen et al., 2018). The reduced redundancy and strengthened complementarity would then enhance resource‐use efficiency, eventually promoting ecosystem functioning (Cadotte et al., 2011; Schmera et al., 2016).

Occasionally, *FEve* has been reported to relate rather poorly with local environment (Liess et al., 2009; Stenger‐Kovács et al., 2019). Assuming that functionally similar species were indeed clustered in space and utilizing the resources without niche overlap as the high observed *FDiv* would imply, the unbalanced resource use should have been reflected in greater irregularity of species abundance distribution in sparsely occupied functional space (Schleuter et al., 2010). However, the environmentally heterogeneous pools may have buffered against complete under‐ or overutilization of resources in the occupied niche space, which would be expected under distinctly lowered levels of *FRic* and *FEve* (Mason et al., 2005).

Variation in *FRic* and *FDiv* was associated with pool spatial location, partly contrasting with our second hypothesis. The degree of pool isolation increased *FRic* and reduced *FDiv*. Isolated rock pools are typically less prone to wind‐mediated invasions by passively dispersing species than pools with permanent watercourse connections (Vanschoenwinkel, Gielen, Seaman, et al., 2008, Vanschoenwinkel, Gielen, Vandewaerde, et al., 2008). Scarcer supply of environmentally highly specialized species might decrease local functional diversity through stronger resource competition between functionally similar species unable to utilize all potentially available resources in the pool environment. As very low degree of isolation enables more frequent dispersal and thus enhanced interchange of species and functional traits, the intensifying competition may result in unbalanced over‐ or underpopulation of the available functional space, irrespective of partial redundancy between species. We also note that the spatial variation in functional diversity may reflect the influence of some unmeasured environmental factors or species interactions such as grazing or competition not quantified here.

Functional divergence further peaked in pools closest to the sea. The majority of the studied pool biota consisted of freshwater species with adaptations to low conductivities, with a growing share of more salinity‐tolerant brackish and marine species along the conductivity gradient (Aarnio et al., 2019). At the opposite ends of the conductivity gradient perpendicular to the seashore, the relative extremity in salinity conditions likely suits fewer functional groups in uneven abundances, leaving parts of the functional space unutilized. Conversely, farther away from the coastline wider selection of niches support relatively more species with various salinity tolerances, balancing the density distribution of functionally different species.

### 4.3 Taxonomic versus functional diversity

Multiple studies have recently demonstrated the greater capacity of functional diversity indices in capturing local environmental variability compared with taxonomic diversity metrics (Aarnio et al., 2019; Colin et al., 2018; Leruste et al., 2018; Stenger‐Kovács et al., 2019). Apart from *FEve*, the taxonomic and functional metrics applied here were highly collinear, yet different factors were responsible for their variation. In highly disturbed habitats, environmental filtering usually limits trait diversity due to functional redundancy, weakening the performance of functional diversity metrics (Mazzei et al., 2018). In our study system, however, this seems unlikely—rather, the moderately low *FEve* refers to functionally divergent communities with ecologically specialized biota dominated by few functional guilds (Cadotte et al., 2011). Possibly, the frequent yet temporary exposure to seawater intrusions may have limited permanent establishment of marine species and hence functional evenness and redundancy in these communities (Mazzei et al., 2018; Mouchet et al., 2010).

Despite the high taxonomic diversity previously discovered for the studied pool communities (Aarnio et al., 2019), our models may also have suffered from insufficiently low trait diversity. The strong dependency of *FRic* on species richness and abundance also makes it highly sensitive to outliers, limiting its applicability on communities with more than 30 species (Laliberté & Legendre, 2010; Mason et al., 2005; Mouchet et al., 2010). Likewise, due to the multitude of traits occasionally exceeding local pool species richness, variation in distribution of ecologically different taxa characterized by various functional strategies may have been lower than required for sufficiently filling the functional space. For example, many life forms are similar in their adaptations to high nutrient levels (Berthon et al., 2011). Alternatively, partly correlated functional guilds (such as colonials and high profile) may have led to underestimated functional diversity in our study system (Mason et al., 2005; Villéger et al., 2008).

Functional richness and evenness were overall moderate and negatively linked to functional divergence. *FRic* is naturally positively correlated with local species richness, yet the causality in this relationship is primarily unidirectional due to functional trait variability (Heino, 2008; Mouchet et al., 2010). Within two taxonomically equally diverse communities, functional diversity may be highly unbalanced, adding to the vulnerability of the functionally less diverse community (Schleuter et al., 2010; Violle et al., 2014). Thus, even rapid or sudden fluctuations in taxonomic diversity rarely affect functional diversity in redundant communities, whereas instability in functional diversity will inevitably be reflected in taxonomic diversity (Cadotte et al., 2011; Schmera et al., 2016).

## 5 CONCLUSIONS

The exact definition and tools for quantification of functional diversity have been open to discussion ever since the functional approaches begin to gain foothold in ecology (Mouchet et al., 2010). Several promising results of the usefulness of the functional diversity metrics have increased the interest toward trait‐based methods in quantifying local biodiversity (Green et al., 2008). While a consensus of the best performing diversity index combination is yet to be reached, those accounting for interspecific variation in ecological adaptations are highly recommended (Schleuter et al., 2010; Villéger et al., 2008).

All diversity metrics except *FEve* were highly collinear; *FEve* was merely significantly linked to *J*. Each of our regression models—whether taxonomic or functional—performed successfully yet rather similarly when explained by the covariates. All six diversity indices were always explained by water conductivity and temperature–sampling month interaction. Taxonomic diversity was further always explained by pool distance from the sea regardless of the index, supporting our hypothesis of their stronger relatedness with spatial processes. Contrary to our expectations, functional diversity indices were related not only to water physicochemistry but spatial factors such as pool isolation as well. In light of our findings, we cannot completely support the generally acknowledged superiority of functional indices over the taxonomic ones in explaining coastal microalgal diversity in a changing environment.

Taken together, the combination of taxonomic and functional indices applied here seems to meet best the criteria for adequately quantifying local diversity. As functional indices inevitably ignore the influence of some interspecific environmental adaptations better captured at the level of individual species, their robustness and ability to reveal complex environmental interactions in communities of unbalanced abundance distribution (Berthon et al., 2011; Cadotte et al., 2011) were successful. However, *FEve* showed partly unique responses to spatiotemporal differences in environmental variation compared with the other diversity indices, and there was a partial inability of the functional indices to reflect declines in taxonomic richness due to redundancy (Mouchet et al., 2010; Schmera et al., 2016).

Despite their small size, biologically complex and diverse rock pools are commonly considered as representatively reflecting community dynamics encountered in larger aquatic systems (De Meester et al., 2005; Srivastava et al., 2004), yet the extent of this reminiscence remains understudied. We therefore conclude that as individual metrics may be driven by different factors, the greatest advantage in assessing local diversity in small‐scale aquatic communities is probably achieved with a simultaneous application of both taxonomic and functional diversity indices.

## CONFLICT OF INTEREST

None declared.

## AUTHOR CONTRIBUTIONS

**Sonja Aarnio:** Formal analysis (lead); writing–original draft (lead); writing–review and editing (equal). **Janne Soininen:** Conceptualization (lead); supervision (lead); writing–original draft (supporting); writing–review and editing (equal).

## Data Availability

The data are available in Dryad repository (https://doi.org/10.5061/dryad.d2547d83b).

## APPENDIX 1

Schematic map showing all sampled rock pools numbered 1–30, and their relative location to each other and to the coastline. The dashed border extending upwards from pool 22 represents an increase in pool area from June onwards. The seashore is located vertically to the left of the pools, marked as a blue solid line.

## APPENDIX 2

Sampling design. Each of the 30 pools was sampled thrice, that is, once a month, resulting in 3 × 30 samples in total. In the generalized linear mixed models, sampling month was treated as a fixed term and sampling site as a random intercept.

## APPENDIX 3

Packages utilized in the statistical analyses conducted with R software. For full references, see References in the main text

**Table:**

Package	Reference
dplyr	Wickham et al. (2021)
FD	Laliberté et al. (2014)
ggplot2	Wickham (2016)
GLMMadaptive	Rizopoulos (2021)
glmmTMB	Brooks et al. (2017)
Hmisc	Harrell (2020)
lattice	Sarkar (2008)
MASS	Venables and Ripley (2002)
mgcv	Wood (2011)
performance	Lüdecke et al. (2020)
pgirmess	Giraudoux et al. (2018)
plyr	Wickham (2011)
vegan	Oksanen et al. (2019)

## APPENDIX 4

Statistics for the explanatory variables. Pool area is calculated by multiplying pool length by pool width; Y coordinates and pool distance from the sea (or X coordinates) are estimated measures from a grid map drawn of the study area. Pool isolation is the average distance from a pool to five geographically closest pools. Note that pool area and Y coordinates were excluded from the statistical analyses

**Table:**

Variable	Min	Max	Mean	Median	*SD*
Total P (μg/L)	17.30	1,448.30	214.00	125.30	255.70
pH	6.7	10.0	–	8.6	0.8
Conductivity (μS/cm)	38	16,741	4,101	1,644	4,384
Temperature (°C)	14.8	26.2	19.7	19.7	1.9
Depth (m)	0.05	0.40	0.20	0.20	0.08
Area (m^2^)^a^	0.09	36.72	3.81	0.94	7.90
Distance to sea (m)^b^	1.6	11.4	6.6	6.9	2.70
Isolation (m)	2.7	18.9	7.7	7.1	4.2
Y coordinate (m)^a^	1.4	106.2	50.5	49.6	28.0

^a^ Variable not included in the statistical analyses.

^b^ That is, X coordinates.

## APPENDIX 5

The Spearman rank correlation coefficients and their statistical significance for the relationship between each variable pair. The statistical significance of the variables is based on the *p* value: – non‐significant, **p* < .05, ***p* < .01, ****p* < .001. Bolded correlation coefficients are statistically significant (*p* < .05)

**Table:**

	Total P	pH	Conductivity	Temperature	Depth	Distance to the sea	Isolation	Month	Site	*S*	*J*	*H*	*FRic*	*FEve*	*FDiv*
Total P		0.19	−0.14	**0.47**	**−0.29**	0.19	−0.04	**−0.27**	−0.04	−0.05	0.07	0.02	−0.02	−0.10	**0.23**
pH	–		**0.22**	0.18	**−0.21**	−0.04	0.08	**−0.64**	−0.09	0.19	**0.26**	**0.25**	−0.05	**−0.26**	−0.03
Cond.	–	*		**−0.29**	−0.13	**−0.52**	−0.05	−0.10	**−0.62**	−0.11	−0.01	−0.02	**−0.40**	−0.20	−0.03
Temp.	***	–	**		**−0.23**	0.04	−0.04	**−0.44**	**0.32**	0.06	**0.23**	0.20	0.11	−0.03	0.08
Depth	**	*	–	*		**0.32**	**−0.27**	**0.21**	−0.02	−0.09	−0.19	−0.18	0.11	0.13	0.11
Distance	–	–	***	–	**		**−0.23**	0.00	0.05	−0.03	**−0.22**	−0.19	0.20	**0.22**	0.01
Isolation	–	–	–	–	**	*		0.01	**0.25**	0.19	**0.22**	**0.22**	0.20	**−0.22**	**−0.32**
Month	**	***	–	***	***	–	–		0.00	**−0.37**	**−0.48**	**−0.49**	−0.11	0.13	−0.06
Site	–	–	***	*	–	–	*	–		**0.25**	0.18	0.21	**0.45**	−0.02	−0.17
*S*	–	–	–	–	–	–	–	***	*		**0.69**	**0.82**	**0.63**	−0.02	**−0.45**
*J*	–	*	–	*	–	*	*	***	–	***		**0.97**	**0.40**	**−0.24**	**−0.44**
*H*	–	*	–	–	–	–	*	***	–	***	***		**0.49**	−0.20	**−0.48**
*FRic*	–	–	***	–	–	–	–	–	***	***	***	***		0.00	**−0.43**
*FEve*	–	*	–	–	–	*	*	–	–	–	*	–	–		0.13
*FDiv*	*	–	–	–	–	–	**	–	–	***	***	***	***	–	

## APPENDIX 6

Spatial and temporal variation of relative occupancy, abundance, and taxa diversity in diatom life form, ecology, and metabolism. For the relative abundance and diversity, monthly means and their range per site (i.e., 30 pools) are shown; the overall occupancy, abundance, and diversity refer to the whole study period (i.e., 90 pools in May–July). Note that the solitary guild was not included in the statistical analyses due to collinearity with the colonial guild

**Table:**

Guild	Occupancy (%)				Abundance (%)				Taxa diversity (%)			
	Overall	May	June	July	Overall	Per site (range) May	Per site (range) June	Per site (range) July	Overall	Per site (range) May	Per site (range) June	Per site (range) July
Mobile	100	100	100	100	84	87 (50–98)	83 (17–99)	81 (0–99)	83	80 (71–89)	79 (68–88)	76 (20–88)
Pedunculate	100	100	100	100	33	32 (3–93)	34 (2–88)	34 (0.6–98)	26	26 (16–43)	26 (14–42)	31 (13–53)
Stalk‐attached	100	100	100	100	25	23 (1–91)	23 (0.2–83)	28 (0.2–95)	17	17 (8–30)	17 (6–29)	19 (5–40)
Colonial	100	100	100	100	10	12 (1–58)	11 (0.9–42)	6 (0.2–36)	20	15 (8–28)	15 (8–27)	16 (4–30)
Solitary	100	100	100	100	90	88 (42–99)	89 (58–99)	94 (64–100)	80	85 (72–92)	85 (73–92)	84 (70–96)
Adnate	100	100	100	100	4	6 (0.2–25)	3 (0.2–17)	4 (0.2–16)	9	14 (5–23)	14 (7–22)	16 (6–38)
Low	100	100	100	100	22	23 (2–78)	21 (0.6–69)	24 (0.8–83)	16	21 (14–32)	20 (12–31)	24 (13–44)
Motile	99	100	100	97	55	56 (7–94)	57 (9–95)	51 (0–97)	53	52 (38–60)	50 (29–63)	46 (0–66)
High	99	100	100	97	18	20 (2–78)	19 (0.8–86)	14 (0–91)	26	20 (12–36)	22 (13–46)	23 (0–53)
Pad‐attached	99	100	100	97	9	9 (1–26)	12 (0.6–46)	6 (0–40)	10	10 (4–17)	11 (5–17)	13 (0–23)
Arbuscular	93	97	87	97	2	2 (0–6)	1 (0–4)	2 (0–19)	2	5 (0–9)	5 (0–15)	6 (0–20)
Nitrogen‐fixer	93	97	87	97	2	1 (0–5)	1 (0–3)	2 (0–17)	3	3 (0–5)	3 (0–7)	5 (0–20)
Planktonic	89	97	93	77	5	1 (0–13)	4 (0–78)	11 (0–99)	6	6 (0–11)	8 (0–21)	7 (0–60)
Pioneer	88	97	90	77	10	12 (0–66)	9 (0–43)	9 (0–55)	2	4 (0–9)	3 (0–6)	3 (0–9)
Acid‐tolerant	76	80	77	70	2	3 (0–21)	2 (0–10)	1 (0–9)	11	6 (0–12)	6 (0–17)	5 (0–15)
Mucuous tubule	72	87	87	43	1	3 (0–14)	1 (0–16)	< 1	3	3 (0–7)	3 (0–8)	2 (0–7)
Zigzag	93	100	100	80	6	6 (0.3–41)	8 (0.4–41)	3 (0–22)	1	3 (0–5)	3 (0–6)	2 (0–7)
Ribbon	58	57	57	60	<1	<1	<1	1 (0–5)	10	2 (0–10)	3 (0–14)	4 (0–17)
Stellate	20	13	23	23	<1	<1	<1	<1	1	<1	1 (0–3)	1 (0–4)
Filament	10	13	10	7	<1	<1	<1	<1	2	<1	<1	<1
Rosette	6	13	0	3	<1	<1	0	<1	1	<1	0	<1

## APPENDIX 7

Temporal (a) and spatial (b) variation in taxonomic (left panel) and functional (right panel) diversity between the 3 months and 30 study sites, respectively. Shown are monthly median (the horizontal black line), lower and upper quartiles (the box vertical limits) and lower and upper 95‐percentiles (the whiskers). The black dots represent the observed values; outliers are falling 1.5‐fold outside the quartiles.

## APPENDIX 8

Fit of the generalized linear mixed model for Shannon's diversity and Pielou's evenness. Shown is the observed (hollow circles) and predicted relationship (fitted Lowess smoothing curves with 95% confidence intervals) for each statistically significant (a) covariate and (b) month–covariate interaction explaining variation in the response variable. Note that the conductivity values are ln‐transformed.

## APPENDIX 9

Fit of the generalized linear mixed models for (a) functional richness and (b) functional divergence. Shown is the observed (hollow circles) and predicted relationship (fitted Lowess smoothing curves with 95% confidence intervals) for statistically significant covariates explaining variation in the response variable. Note that some of the covariate values are ln‐transformed.
